# An Active Retirement Programme, a Randomized Controlled Trial of a Sensorimotor Training Programme for Older Adults: A Study Protocol

**DOI:** 10.3390/healthcare11010086

**Published:** 2022-12-28

**Authors:** Carolina Alexandra Cabo, Orlando Fernandes, María Mendoza-Muñoz, Sabina Barrios-Fernandez, Laura Muñoz-Bermejo, Rafael Gómez-Galán, Jose A. Parraca

**Affiliations:** 1 Departamento de Desporto e Saúde, Escola de Saúde e Desenvolvimento Humano, Universidade de Évora, Largo dos Colegiais 2, 7000-645 Évora, Portugal; 2 Comprehensive Health Research Centre (CHRC), University of Évora, Largo dos Colegiais 2, 7000-645 Évora, Portugal; 3 Research Group on Physical and Health Literacy and Health-Related Quality of Life (PHYQOL), Faculty of Sport Sciences, University of Extremadura, 10003 Caceres, Spain; 4 Occupation, Participation, Sustainability and Quality of Life (Ability Research Group), Nursing and Occupational Therapy College, University of Extremadura, 10003 Caceres, Spain; 5 Social Impact and Innovation in Health (InHEALTH), University Centre of Mérida, University of Extrema dura, 06800 Mérida, Spain; 6 Research Group on Physical and Health Literacy and Health-Related Quality of Life (PHYQOL), University Centre of Mérida, University of Extremadura, 06800 Mérida, Spain

**Keywords:** elderly, falls, gait, postural control, exercise, quality of life

## Abstract

Research shows that exercise training programmes lead to several improvements in older adults’ health-related quality of life (HRQoL) and well-being. This study will examine the effects of an active retirement programme on Portuguese older adults, investigating its effects on body composition, physical fitness, HRQoL, and physical activity level (PAL). Therefore, a parallel-group randomised controlled trial will be conducted, including body composition (height and body weight), physical fitness (strength, flexibility, agility, postural control, and gait), HRQoL, and PAL assessments before and after the application of the programme. The programme will be carried out for six months, two days per week (45 min), plus a year of follow-up. The programme will consist of six circuits with eight physical exercises each. The circuits will change at the end of the four weeks (one monthly circuit). The exercises’ difficulty will increase throughout the programme, with alternatives for all the participants. If the effectiveness of the programme is demonstrated, implementation in different services and municipalities could be advised, as the actors involved in health and social services should promote the well-being of their citizens through, among others, health-related physical activity and the prevention of diseases associated with inactivity.

## 1. Introduction

Demographic ageing is the paradigm of the 21st century, and the older adult population is trending upwards. According to the central forecast scenario of the Office of Statistics National Institute, the number of older adults will increase from 2.1 to 2.8 million between 2015 and 2080 [1]. Thus, understanding and preventing disability in older adults is an important public health challenge, as ageing is a risk factor contributing to functional decline [2]. Ageing is continuous and irreversible and can be related to physical and cognitive function decline [3].

Physical activity (PA) plays an important role in healthy ageing, extending life, and quality of life, reducing chronic the incidence of physical and mental diseases. Moreover, every additional 15 min of daily PA (up to 100 min/day) reduces the mortality rate from any cause 4%. Although mechanisms underlying PA’s positive effects on health have not been fully revealed, evidence points to inflammatory markers, cellular oxidative stress regulation, and the impact on telomere length that could delay the ageing process [4]. According to the World Health Organization (WHO)’s recommendations [5], more than a quarter of the worldwide population (27.5%) does not perform at least 150 min of moderate or 75 min of vigorous PA per week [6]. Older adults should conduct, at least 150 min of moderate-intensity aerobics, at least 75 min of vigorous-intensity aerobics, or an equivalent combination of moderate- and vigorous-intensity PA throughout the week for substantial health benefits. Older adults should also perform moderate or greater-intensity muscle-strengthening activities that involve all major muscle groups two or more days a week, as these provide additional health benefits. As part of their weekly PA, older adults should practise multicomponent PA, emphasising functional balance and strength training at a moderate or high intensity three or more days a week to prevent falls [5]. In this sense, Gopinath et al. [4] carried out a study for 10 years in Australia, following up with adults over 50 years involved in moderate-vigorous PA (≥ 5000 MET minutes/week), which found that participants doubled their survival probability without chronic diseases, cognitive deficits, or functional incapacity. Moreover, PA levels (PAL) in older people should be higher than those recommended by the WHO (600 MET minutes/week) [5] to reach healthy ageing. There are 2.4 million individuals aged 65 and over in Portugal [7], with only 16.6% following the WHO’s recommendations [8]. A lack of PA contributes to the loss of functional fitness in the elderly [9]. Aerobic exercise is important for optimal musculoskeletal performance; however, aerobic capacity gradually declines with advancing age. Likewise, exercised muscles are more sensitive to anabolic stimuli, such as protein ingestion, which allow muscle protein synthesis [10]. As a result, sedentary elderly people have lower muscle mass and reduced bone mass, and, consequently, their ability to perform PA is affected.

Psychosocial factors also impact the functional status of the elderly. Self-efficacy and fear of falling are important factors [11]. Self-efficacy is not only associated with gait speed and physical-function limitations but also with participation in physical exercise programmes. In turn, fear of falling negatively influences sedentary behaviour time. Other psychosocial factors associated with reduced functional fitness are loneliness, depression, and exhaustion [10]. Moreover, physical factors impact the ageing process. A decrease in strength leads to a lower functional capacity, loss of balance, and decreased gait speed [12]: older adults generally have a wider gait, postural sway, and greater gait variability when walking [13]. Moreover, the sensorimotor system incorporates all processing and the afferent, efferent, and centrally integrating components involved in joints’ functional stability. As people age, this ability becomes limited, leading to important biomechanical changes in gait [14]. Loss of mobility, decreased muscle strength, and a balance deficit all contribute to dependence [15]. Hence, sensorimotor training improves older adults’ balance and provides gait confidence, although it does not affect the biomechanical parameters [16]. Several studies on sensorimotor training reported improvement in performance in clinical balance and mobility [17]: sensorimotor training-induced shortened onset latency in postural muscles [18], decreased onset latency, and enhanced reflex activity [19]. Sensorimotor training also induces an improvement in maximal and explosive-force production of leg extensors’ capacity [20] and improves lower-limb strength and power, static and dynamic balance, and mobility [21].

Research on how PA affects health-related quality of life (HRQoL) has shown that PA’s benefits regarding quality of life were limited [22,23]. Several studies have evaluated the sensorimotor system in the elderly and obtained strength, mobility, and balance improvements, but these results were not consistent due to the reduced sample [20,21], being short-term (12 weeks or less), or only assessing some abilities [17]. This study will last 24 weeks, and its effects will be measured through gait, postural control, strength, flexibility, balance, and agility. Hence, this study will aim to (1) assess the effectiveness of an active retirement programme on elderly body composition, physical fitness, HRQol, and PAL and (2) assess the programme’s effectiveness throughout the ageing process (follow-up).

## 2. Materials and Methods

### 2.1. Design

A parallel-group randomised controlled trial will be conducted, including a 6-month intervention phase and a 1-year follow-up period. For both groups (control and experimental), assessments will be performed at baseline (before starting the intervention), and after the intervention ends. The study will follow the Consolidated Standards of Reporting Trials Statement (CONSORT) [24].

### 2.2. Ethics

The Ethics Committee of the University of Évora approved this project (approval number: 21040). The study was registered with the Clinical Tri-als.gov PRS Protocol Registration and Results System (Registration Number: NCT05398354; https://www.clinicaltrials.gov/ct2/show/NCT05398354?term=NCT05398354&draw=2&rank=1 (accessed 12 June 2022).

### 2.3. Sample Size

Sample size calculations were performed using the G*Power 3.1.9.4 software (Kiel University, Kiel, Germany), selecting the statistical test to compare the difference between two independent means (two groups). Thus, accepting an alpha risk of 0.05 and a beta risk of 0.2 in a bilateral contrast and assuming a moderate effect size of 0.5, a total of 160 participants (80 subjects in the experimental group and 80 in the control group) were sufficient to reach a minimum potency of 90%.

### 2.4. Randomisation and Blinding

Participants will be randomly assigned to the experimental (active retirement programme) or control groups. To assign participants to each group (1:1), Research Randomizer software (version 4.0, Geof-frey C. Urbaniak and Scott Plous, Middletown, CT, USA; http://www.randomizer.org, accessed 12 June 2022) will be used to create a randomisation sequence. A member of the research team, who will not participate in the intervention, will carry out this process. Group assignment will be hidden in a password-protected computer file. Participants will know their group assignment, but outcome assessors and data analysts will not know the participants’ group assignment.

### 2.5. Participants

Participants must meet the following inclusion criteria: (1) be retired; (2) be aged between 55 and 80; (3) show their agreement to participate in the study by providing a signed consent form; (4) be individuals without dentures (except dental prosthesis); and (4) not have undergone surgery for less than six months. Exclusion criteria will be individuals with (1) musculoskeletal diseases; (2) locomotion issues; (3) psychiatric disorders; (4) neurological disorders; and (5) cardiovascular diseases.

### 2.6. Intervention

**Figure 1:**
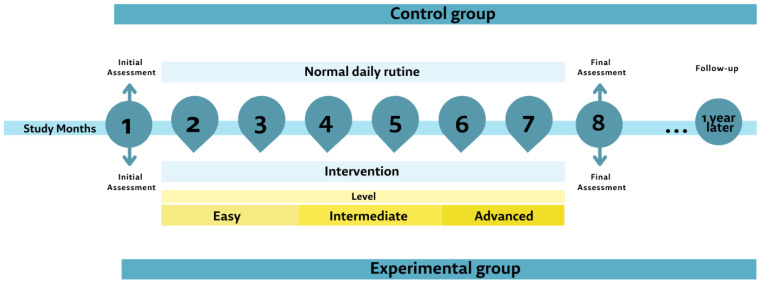
Study timeline graph.

**Experimental group:** This sensorimotor training programme will be carried out for six months, twice per week. As shown in Figure 1, as the programme progresses the load will be progressively increased.

**Figure 2:**
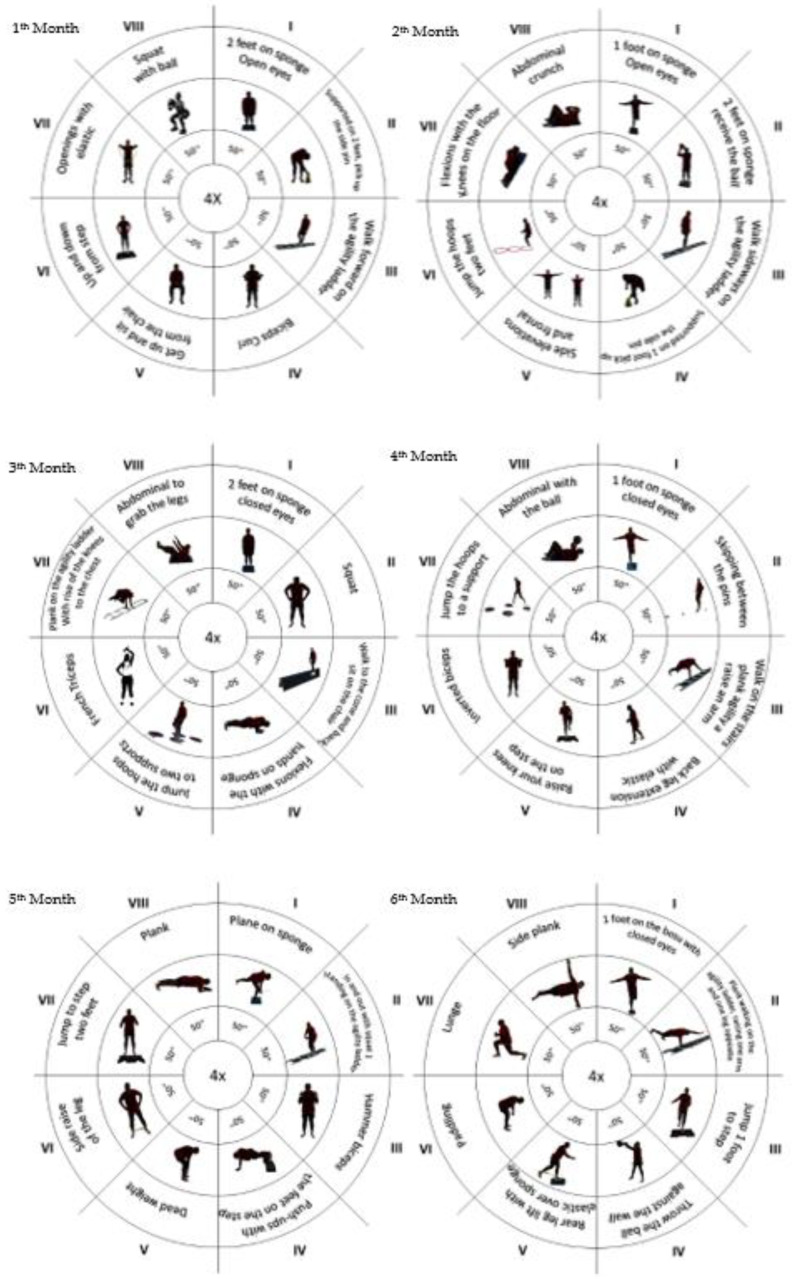
Sessions’ main part planning.

Sessions will be divided into three intensity levels: easy (no external load during the first eight weeks), intermediate (application of external load: elastic bands, shin guards, and free weights, from the 9th week to the 16th week) and advanced (increase in external load for the previous level, from the 17th week to the 24th week). Each month, a different type of session will be developed (Figure 2). Each session will last 45 min and will be divided into three phases: an initial one, consisting of a 5 min walk followed by a joint warm-up (10 min); a fundamental phase (25 min), working on exercises circuits consisting of four cycles, with eight exercises each (50 s on and 15 s off); and a return to calm with muscle stretching (10 min). Additionally, at the end of every session, the intensity will be assessed using the Borg Rating of Perceived Exertion Scale, and the level of satisfaction of the participants will be assessed employing the Physical Activity Enjoyment Scale (PACES) [24].

**Control group:** Individuals will continue with their normal daily routine, only participating in the assessments. They will be offered to perform the exercise programme when the study ends.

Moreover, to guarantee participants’ safety, different strategies will be applied:

- Anamnesis of all participants;
- Hygiene of the material to be used during the sessions;
- Breaks between exercises;
- More than one researcher assisting with the sessions;
- Application of the effort scale.

### 2.7. Measures

**Figure 3:**
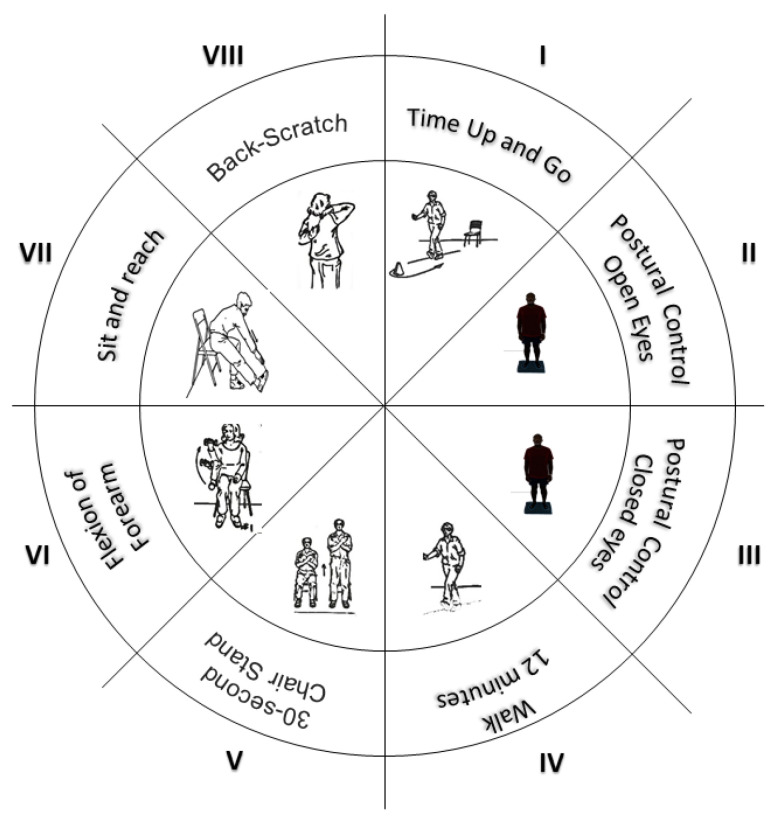
Assessments for physical fitness measurements.

A variety of tools will be used. All measures will be undertaken at baseline, at the end of the intervention, and one year after the end of the intervention (follow-up). Before the first measurement, all participants will go through a familiarisation phase to familiarise themselves with the different instruments and assessments included in this project.

a. Anthropometrics and body composition. Bodyweight and height will be assessed. Before the measurements, participants will be asked to remove their shoes, socks, and heavy clothing (coats, sweaters, coats, etc.). They will also be asked to empty their pockets and remove belts and other accessories (bands, pendants, etc.). Height will be measured using a stadiometer (Seca 22, Hamburg, Germany). This instrument must be placed on a vertical surface with the measuring scale perpendicular to the ground. Participants will be positioned in a standing position, with their shoulders balanced, and their arms relaxed along their body. Height will be taken in cm and rounded to the nearest mm. Body weight will be measured using a scale. Body weight will be recorded in kg. and the body mass index (BMI) will be determined using the formula: weight × height^2^.

b. Physical fitness. Participants will wear tracksuit bottoms and will be asked to remove accessories and any objects in their pockets. The following measures will be carried out (Figure 3): (I) agility and execution speed will be assessed through the Timed Up and Go (TUG) test, which consists of getting up from a chair, walking in a straight line three meters away, and walking back and sitting down again [25]; (II and III) postural control will be tested through a force platform (Ber-tec4060-Columbus; USA). The assessment will consist of measuring the oscillations in a static bipedal position, with eyes open (two minutes) and with eyes closed (two minutes) [26]; (IV) gait will be assessed using the mobile application “Phyphox” on the surface of the skin, at the inner edge of the tibia, to quantify the number of steps and time. Participants will be asked to walk a pre-established route, without slopes or obstacles, for 10 min, at their natural cadence and, later, walk the same route at a pace determined by complex stimuli (auditory metronome—loudspeakers, which allow for hearing beats that correspond to steps) [27]; (V) muscular endurance will be assessed by rising up from the chair or bending and straightening for 30 s, during which the strength of the lower limbs involving the vastus medialis obliquus (VMO) and the vastus lateralis (VL) will also be calculated [28]; (VI) upper limb strength will be determined by the number of times that a weight can be lifted by performing a flexion–extension of the arms for 30 s [28]; (VII and VIII) flexibility will be evaluated by two tests: “the sit and reach” for lower limb flexibility, where participants, from a seated position with one leg extended, will slowly bend down, sliding their hands down the extended leg until they touch (or pass) their toes [28]; and “the behind the back reach” test for upper limbs, assessing the shoulder’s full range of motion, which will consist of measuring with a ruler the distance between (or the overlap of) the middle fingers behind the back [28].

c. HRQoL. This will be assessed using the 36-Item Short-Form Survey (SF-36) [29] in its Portuguese version [30,31], a 36-question tool, which results in 8 dimensions of health status (physical function, physical role, bodily pain, general health, vitality, social function, emotional role, and mental health) and 2 summary components (physical and mental). Dimensions and components are scored from 0 to 100, where 0 is the worst state, and 100 is the best.

d. PAL. This will be assessed using the International Physical Activity Questionnaire Short Form (IPAQ-SF) [32]. This instrument consists of four questions informing on the frequency (days/week) and duration (minutes/day) of daily walks and activities requiring moderate to vigorous physical exertion, as well as the time (minutes/day) spent on sitting activities on weekdays and weekends. PA will be classified into three categories according to the IPAQ consensus group: sedentary (< 600 Met-minutes/week), active (≥ 600 Met-minutes/week), and very active (≥ 3000 Met-minutes/week). This instrument will be completed by the participants in its Portuguese version [33].

e. Subjective Perception of Effort. This will be assessed through the Borg Rating of Perceived Exertion Scale [34] during the sessions, consisting of 10 items for which participants rate their effort from “not at all” (1) to “maximum” (10).

f. Adherence rate. This will be controlled. For this purpose, following previous studies [35,36] the following parameters will be monitored: proportion of participants completing exercise programmes, proportion of exercise sessions attended, average number of exercise sessions completed per week, class attendance expressed as a proportion of participants reaching certain cutoffs, number of weeks in which exercise was undertaken, and proportion of days on which exercise was undertaken. In addition, recommendations from previous studies will be followed to encourage adherence to the programme, including making instructions to subjects simpler and less demanding, addressing cognitive motivational factors such as self-efficacy and health beliefs, offering social support and reinforcement, and providing reminders [37,38].

g. Level of satisfaction. This will be evaluated through the Physical Activity Enjoyment Scale (PACES) survey, which consists of 8 items that are scored from 1 to 7 points (where 1 = “I enjoy it”, 7 = “I hate it”, and 4 = “neutral”). The total score will be calculated from the sum of the items, with a maximum possible score of 56 points and a minimum of 8; the higher the score is, the greater the enjoyment is. The Portuguese version will be completed by the participants [24].

### 2.8. Statistical Analysis

Descriptive statistics and computations will be performed with SPSS (version 25.0; IBM SPSS Inc., Armonk, IL, USA). Personal data will be kept anonymous.

The normality and homogeneity of data will be checked by applying Kolmogorov– Smirnov and Levene’s tests, respectively. Data will be presented as means and standard deviation (SD) (parametric variables) or median and interquartile range (IR) (nonparametric variables). The independent samples t-test (parametric variables) or the Mann–Whitney U test (non-parametric variables) will be used to determine whether the experimental and control groups were comparable at baseline in terms of participant characteristics. Then, repeated measures of ANCOVA will be applied to analyse the intervention effects on the different dependent variables, adjusted by age and baseline outcomes. Cohen’s d (with a 95% confidence interval) will also be included in the results as the effect size. Effect size thresholds will be interpreted as follows: >0.2, small; >0.5, moderate; >0.8, large [36]. Statistical significance will be computed for the effect of time and the interaction group × time. The alpha level will be fixed at *p* ≤ 0.05.

## 3. Discussion

This project will be the first conducted developing a sensorimotor training programme for 24 weeks using different sensorimotor skills in Portuguese older adults, to test this programme’s effects on HRQoL and fall prevention compared to usual care. Different difficulty levels will be included, facilitating individualisation. This training programme can be applied by multiple agents who might be interested in reaping the benefits of these types of activities, since this training method does not require any specific installation and can be completed indoors or outdoors.

In this sense, Avelar et al. [21] carried out a similar quasi-experimental study, with a similar training period composed of exercise circuits to improve strength, gait, functional reach, and static and dynamic balance, among others. Positive responses in lower limb strength, power, mobility, and static and dynamic balance were found, although the sample was small [20,21,27], compared to the 160 participants expected for our study. Other research [17,18,39] used sensorimotor training programmes on functional capacity and balance for a short time, from two to six weeks. Despite the short period, positive results were obtained concerning balance, mobility, activities of daily living performance, and fall risk. Thus, by improving physical capabilities, fall risk could be lower. Other studies [19,20,21] have applied 12–13 weeks of sensorimotor training, half of what is proposed in this protocol. There were positive results for mobility and balance and an improved ability to produce the maximum and explosive force of leg extensors. Several of these studies developed a short-time practical application [39], had a small size [21], or assessed only one or two sensorimotor abilities [17,18,19,20]. For all these reasons, no studies with consistent results have produced an advance on this topic, bearing in mind that the percentage of old people practising PA is very low [40], so it is essential to promote PA practise, as sensorimotor skills may be deficient.

The application of this study is expected to obtain an increase in physical capacities such as strength, flexibility, gait, postural control, balance, and agility. In addition, we are also aiming to improve the participants’ HRQoL and reduce their fall risk. The programme could be applied to public and private entities. In the public sector, the application of active retirement programmes within the services offered by public health programmes exists at a regional level, as is the case of the “Alma Senior” programme, developed in the Municipality of Almada, Portugal, or the “The Exercise Looks After You” programme, developed in Extremadura, Spain, which increases its cost–benefit ratio for all aspects related to HRQoL and fall prevention in this sector. Likewise, the possibility of implementing this training system in different associations can be studied. In the private sector, a focus group study can include the heads of health and sports centres, focusing on the applicability of sensorimotor training in their centres, with the results of the study able to highlight the potential advantages of private sector application.

The Portuguese population has experienced a substantial increase in average life expectancy in the last four decades. During the same period, there has been a progressive increase in health expenditure [41]. Regarding the relationship between ageing and health expenditure, there does not seem to be a consensus in the literature on the effects of ageing on health expenditure. The literature refutes that population age is a major determinant of health expenditure and other factors, thus considering a strong positive correlation between the two variables. This correlation is not confirmed in the majority of econometric analyses that have been developed in the last two decades in the area of health economics [42]. Thus, PA should be considered an essential item for public health, since, if performed regularly, it is considered an important condition for health promotion and the prevention/remediation of chronic diseases, for different age groups [43].

Regarding limitations, one of the limitations that will exist for the application of this study is being after/during the COVID-19 pandemic. We must take extra care, by adding space and material for the hygienists, to give greater security to the programme. Travel to the space where the programme will be carried out may also be a limitation, considering that the population under study involves people who no longer travel by car; it may be too far away for them to travel on foot, so there will be increased costs due to the necessity of using public transport.

In the future, it would be interesting to evaluate all age groups, in addition to this age group, such as those who are still actively employed, to have a better perception of what happens throughout life in terms of sensorimotor behaviour, not just during the ageing process. This could result in people being able to act from an early age, to improve the capacities analysed in this study. In addition, it would be interesting to add other clinical variables more related to health, such as blood pressure and cholesterol, because, as reported in other studies [44], there were improvements in these variables in the physical condition of participants. We can also analyse the effects of the programme on the number of medical appointments. Finally, applying a pre-participation physical assessment of the participants by a sports physician would be beneficial.

The added value of this study will be the acquired knowledge about sensorimotor training for the elderly, which will present a scientific advancement for PA, HRQoL, and sedentary behaviour prevention. Finally, it will be innovative in creating an intervention manual and a digital library with specific exercises for sensorimotor training, which allow for better adaptation and motor coordination.

## 4. Conclusions

This study will investigate the effectiveness of an active retirement programme in the elderly on their body composition, physical condition, HRQoL, and fall risk, advancing the knowledge of PA, health, and wellness.

## Data Availability

Not applicable.

## References

[1] Instituto Nacional de Estadística. https://www.ine.pt/xportal/xmain?xpgid=ine_main&xpid=INE.

[2] Anton S.D., Cruz-Almeida Y., Singh A., Alpert J., Bensadon B., Cabrera M., Clark D.J., Ebner N.C., Esser K.A., Fillingim R.B. (2020). Innovations in Geroscience to Enhance Mobility in Older Adults. Exp. Gerontol..

[3] Wong T.K.K., Ma A.W.W., Liu K.P.Y., Chung L.M.Y., Bae Y.-H., Fong S.S.M., Ganesan B., Wang H.-K. (2019). Balance Control, Agility, Eye–Hand Coordination, and Sport Performance of Amateur Badminton Players: A Cross-Sectional Study. Medicine.

[4] Gopinath B., Kifley A., Flood V.M., Mitchell P. (2018). Physical Activity as a Determinant of Successful Aging over Ten Years. Sci. Rep..

[5] Bull F.C., Al-Ansari S.S., Biddle S., Borodulin K., Buman M.P., Cardon G., Carty C., Chaput J.-P., Chastin S., Chou R. (2020). World Health Organization 2020 guidelines on physical activity and sedentary behaviour. Br. J. Sport. Med..

[6] Guthold R., Stevens G.A., Riley L.M., Bull F.C. (2018). Worldwide trends in insufficient physical activity from 2001 to 2016: A pooled analysis of 358 population-based surveys with 1.9 million participants. Lancet Glob. Health.

[7] PORDATA (2021). População Residente Com 65 E Mais Anos, Estimativas a 31 de Dezembro: Total E Por Grupo Etário.

[8] (2021). Portugal Physical Activity Factsheet. https://cdn.who.int/media/docs/librariesprovider2/country-sites/physical-activity-factsheet---portugal-2021.pdf?sfvrsn=d8fac7be_1&download=true.

[9] Ghram A., Briki W., Mansoor H., Al-Mohannadi A.S., Lavie C.J., Chamari K. (2021). Home-Based Exercise Can Be Beneficial for Counteracting Sedentary Behavior and Physical Inactivity during the COVID-19 Pandemic in Older Adults. Postgrad. Med..

[10] Tieland M., Trouwborst I., Clark B.C. (2018). Skeletal Muscle Performance and Ageing: Skeletal Muscle Performance and Ageing. J. Cachexia Sarcopenia Muscle.

[11] Garcia Meneguci C.A., Meneguci J., Sasaki J.E., Tribess S., Júnior J.S.V. (2021). Physical Activity, Sedentary Behavior and Functionality in Older Adults: A Cross-Sectional Path Analysis. PLoS ONE.

[12] Brief A. (2012). Envelhecimento, força muscular e atividade física: Uma breve revisão bibliográfica. Rev. Científica FacMais..

[13] Ready E.A. (2019). Optimizing Gait Outcomes in Parkinson’s Disease with Auditory Cues: The Effects of Synchronization, Groove, and Beat Perception Ability.

[14] Suzuki K., Niitsu M., Kamo T., Otake S., Nishida Y. (2019). Effect of Exercise with Rhythmic Auditory Stimulation on Muscle Coordination and Gait Stability in Patients with Diabetic Peripheral Neuropathy: A Randomized Controlled Trial. OJTR.

[15] Bacha J.M.R., Cordeiro L.R., Alvisi T.C., Bonfim T.R. (2016). Impacto Do Treinamento Sensório-Motor Com Plataforma Vibratória No Equilíbrio e Na Mobilidade Funcional de Um Indivíduo Idoso Com Sequela de Acidente Vascular Encefálico: Relato de Caso. Fisioter. Pesqui..

[16] Rezende A.A.B., Silva I.L., Beresford H., Batista L.A. (2012). Avaliação Dos Efeitos de Um Programa Sensório-Motor No Padrão Da Marcha de Idosas. Fisioter. mov..

[17] Steadman J., Donaldson N., Kalra L. (2003). A randomized controlled trial of an enhanced balance training program to improve mobility and reduce falls in elderly patients. J. Am. Geriatr. Soc..

[18] Hu M.-H., Woollacott M.H. (1994). Multisensory Training of Standing Balance in Older Adults: I. Postural Stability and One-Leg Stance Balance. J. Gerontol..

[19] Granacher U., Gollhofer A., Strass D. (2006). Training induced adaptations in characteristics of postural reflexes in elderly men. Gait Posture.

[20] Granacher U., Gruber M., Gollhofer A. (2009). Resistance Training and Neuromuscular Performance in Seniors. Int. J. Sports Med..

[21] Avelar B.P., Costa J.N.D.A., Safons M.P., Dutra M.T., Bottaro M., Gobbi S., Tiedemann A., de David A.C., Lima R.M. (2016). Balance Exercises Circuit Improves Muscle Strength, Balance, and Functional Performance in Older Women. AGE.

[22] De Oliveira A.C., Oliveira N.M.D., Arantes P.M.M., Alencar M.A. (2010). Qualidade de Vida Em Idosos Que Praticam Atividade Física—Uma Revisão Sistemática. Rev. Bras. De Geriatr. E Gerontol..

[23] Marquez D.X., Aguiñaga S., Vásquez P.M., Conroy D.E., Erickson K.I., Hillman C., Stillman C.M., Ballard R.M., Sheppard B.B., Petruzzello S.J. (2020). A Systematic Review of Physical Activity and Quality of Life and Well-Being. Transl. Behav. Med..

[24] Teques P., Calmeiro L., Silva C., Borrego C. (2020). Validation and Adaptation of the Physical Activity Enjoyment Scale (PACES) in Fitness Group Exercisers. J. Sport Health Sci..

[25] Gerhardy T., Gordt K., Jansen C.-P., Schwenk M. (2019). Towards Using the Instrumented Timed Up-and-Go Test for Screening of Sensory System Performance for Balance Control in Older Adults. Sensors.

[26] Salehi R., Ebrahimi-Takamjani I., Esteki A., Maroufi N., Parnianpour M. (2010). Test-Retest Reliability and Minimal Detectable Change for Center of Pressure Measures of Postural Stability in Elderly Subjects. Med. J. Islam. Repub. Iran.

[27] Malacrida J.P. (2021). Aplicativos Em Smartphones: O Despertar Científico No Estudo de Energia. Dissertação de Mestrado.

[28] Rikli R.E., Jones C.J. (1999). Development and Validation of a Functional Fitness Test for Community-Residing Older Adults. J. Aging Phys. Act..

[29] Ware J.E. (1993). SF-36 Health Survey: Manual and Interpretation Guide.

[30] Ferreira P.L. (2000). Development of the Portuguese version of MOS SF-36. Part II—Validation tests. Acta Med. Port..

[31] Ferreira P.L. (2000). Development of the Portuguese version of MOS SF-36. Part I. Cultural and linguistic adaptation. Acta Med. Port..

[32] Lee P.H., Macfarlane D.J., Lam T.H., Stewart S.M. (2011). Validity of the international physical activity questionnaire short form (IPAQ-SF): A systematic review. Int. J. Behav. Nutr. Phys. Act..

[33] Cruz J., Jácome C., Morais N., Oliveira A., Marques A. (2018). Concurrent validity of the Portuguese version of the Brief physical activity assessment tool. BMC Health Serv. Res..

[34] Borg G. (1998). Borg´s Perceived Exertion and Pain Scales.

[35] Pavey T., Taylor A., Hillsdon M., Fox K., Campbell J., Foster C., Moxham T., Mutrie N., Searle J., Taylor R. (2012). Levels and Predictors of Exercise Referral Scheme Uptake and Adherence: A Systematic Review. J. Epidemiol. Community Health.

[36] Picorelli A.M.A., Pereira L.S.M., Pereira D.S., Felício D., Sherrington C. (2014). Adherence to Exercise Programs for Older People Is Influenced by Program Characteristics and Personal Factors: A Systematic Review. J. Physiother..

[37] Flegal K., Kishiyama S., Zajdel D., Haas M., Oken B. (2007). Adherence to Yoga and Exercise Interventions in a 6-Month Clinical Trial. BMC Complement. Altern. Med..

[38] Rivera-Torres S., Fahey T.D., Rivera M.A. (2019). Adherence to Exercise Programs in Older Adults: Informative Report. Gerontol. Geriatr. Med..

[39] Gomes C.S., Rangel G.M.B., Sant’Ana M.E.G.D.S., Dos Santos M.A.T., Fraga W.L.D.A., Soares E.V., Ribeiro Junior S.M.S. (2018). Efeitos do treinamento sensório motor por meio de dispositivos ecoeficientes sobre a capacidade funcional e equilibrio em idosos: Ensaio clinico controlado. BS.

[40] World Health Organization (2021). 2021 Physical Activity Factsheets for the European Union Member States in the WHO European Region.

[41] Santana P., Almendra R. (2018). The Health of the Portuguese over the Last Four Decades: Comments on the Course Taken. Méditerranée. Rev. Géographique Des Pays Méditerranéens J. Mediterr. Geogr..

[42] Estevens J.P.G. (2015). Envelhecimento E Despesa Em SaúDe: O Caso Português. Ph.D. Thesis.

[43] Elesbão H., Ramos E.R., Da Silva J.O., Borfe L. (2021). A Influência Da Atividade Física Na Promoção Da Saúde Em Tempos de Pandemia de Covid-19: Uma Revisão Narrativa. RIPS.

[44] Britto L.S., Vasconcelos Filho F.S.L. (2021). Análise da relação de parâmetros imunológicos com o desenpenho de idoso na corrida. Educação Física Para Grupos Especiais: Exercício Físico Como Terapia Alternativa Para Doenças Crônicas.

